# Validation of a New Stress Induction Protocol Using Speech Improvisation (IMPRO)

**DOI:** 10.3390/brainsci15050522

**Published:** 2025-05-19

**Authors:** Marina Saskovets, Mykhailo Lohachov, Zilu Liang

**Affiliations:** 1 Ubiquitous and Personal Computing Lab, Faculty of Engineering, Kyoto University of Advanced Science, Kyoto 615-8577, Japan; liang.zilu@kuas.ac.jp; 2 Department of Civil and Environmental Engineering, Institute of Science Tokyo, Tokyo 152-8552, Japan; lohachov.m.aa@m.titech.ac.jp

**Keywords:** acute stress induction, electrodermal activity, salivary cortisol, speech improvisation, autonomic nervous system, cognitive stress

## Abstract

**Background:** Acute stress induction is essential in psychology research for understanding physiological and psychological responses. In this study, ‘acute stress’ refers to a short-term, immediate stress response—distinct from chronic, long-term stress exposure. Traditional methods, such as the Trier Social Stress Test (TSST), have ecological validity and resource-efficiency limitations. This study introduces the Interactive Multitask Performance Response Observation (IMPRO) protocol, a novel stress-induction method utilizing speech improvisation in a dynamic and unpredictable social setting. **Methods:** Thirty-five healthy adults (aged 18–38 years; 19 males, 16 females) participated in the study. The IMPRO protocol consisted of three speech improvisation tasks with increasing cognitive and social stressors. Salivary cortisol was used as a biochemical marker of acute stress, while electrodermal activity (EDA) provided real-time autonomic arousal measurements. Stress responses were assessed using paired *t*-tests for cortisol levels and repeated-measures ANOVA for EDA variations across experimental stages. **Results:** Salivary cortisol levels significantly increased from baseline (M = 2.68 nM, SD = 0.99) to post-task (M = 3.54 nM, SD = 1.25, *p* = 0.001, Cohen’s d = 0.59), confirming hypothalamic–pituitary–adrenal (HPA) axis activation. EDA showed a significant rise during the anticipation phase (*p* < 0.001), peaking at the final task and decreasing during recovery (η^2^ = 0.643). **Conclusions:** The IMPRO protocol effectively induces acute stress responses, providing a scalable, ecologically valid alternative to traditional stress paradigms. Its low-cost, adaptable design makes it ideal for research in psychology, neuroscience, and behavioral sciences. Future studies should explore its application in clinical populations and group settings.

## 1. Introduction

Acute stress is an important area of research in psychology due to its profound impact on both mental and physical health [1]. Understanding the mechanisms of stress and how it affects behavior, cognition, and overall well-being is essential for developing effective interventions and treatment strategies. Stress is well known for its neurobiological and biochemical effects. Since the autonomic nervous system regulates internal organs, the stress response engages the entire body through sympathetic activation, leading to increased cortisol levels, elevated blood glucose, and other physiological changes [2]. As Robert Sapolsky noticed, our stress response, initiated by cognitive or social triggers rather than acute survival needs, leads to higher risks of chronic diseases since they are not adapted for permanent activation [3]. As a result, developed societies face a paradox in which biological stressors such as hunger and physical threats have declined, yet stress-related disorders continue to rise. Goodman et al. emphasize the need to expand stress research beyond environmental physiology to include perceptual and psychosocial stressors, which have become increasingly relevant in today’s complex social environments [4].

The development of diverse acute stress-induction protocols is essential for capturing the multifaceted nature of stress, which varies across physiological, cognitive, and environmental dimensions. Wezyk et al. emphasize the diversity in definitions of stress, highlighting that it arises from various factors, including cognitive demands, social pressures, financial strain, and interpersonal challenges, each engaging distinct yet interconnected neuroendocrine pathways. While experts emphasize the hypothalamic–pituitary–adrenal (HPA) axis activation and indirect behavioral effects involved, they also acknowledge the importance of subjective experiences in shaping stress responses [5]. Given the variability in individual responses and the influence of both intrinsic and extrinsic factors, Raggi et al. suggest incorporating cognitive, audiovisual, and speech-based tasks to enhance the ecological validity and cross-study comparability of experimental paradigms [6]. By refining and expanding stress-induction approaches, researchers can improve theoretical stress models, optimize intervention strategies, and enhance the translational relevance of findings to real-world stressors.

Traditionally, researchers have used laboratory-based methods, such as the Trier Social Stress Test (TSST), to induce acute stress in participants. The TSST is well-established for its effectiveness in eliciting a stress response through public speaking in the setting of a mock job interview [7,8]. While variations, such as the TSST for Groups (TSST-G), offer reliable methods for studying acute stress, their ecological validity is limited, as they may not fully capture the complexity of real-world experiences across diverse contexts [9]. Additionally, the TSST does not consistently induce acute stress across all individuals due to factors such as differences in stress resilience or the ability to cognitively disengage from the stressor [10].

Moreover, while the structured nature of the TSST effectively targets social evaluation stressors, it lacks the unpredictability necessary to fully mirror a certain cluster of communicational scenarios. It is also resource-intensive and logistically demanding, limiting its feasibility for field studies. To address these limitations, alternative methods like the Simple Singing Stress Procedure have been introduced to diversify performance-based acute stress induction [11].

Among these, the Sing-a-Song Stress Test (SSST) and its variations in anticipatory singing tasks come closest to reflecting real-life unpredictability. These protocols provoke acute stress responses by unexpectedly instructing participants to sing a song, making them both effective and cost-efficient stress-induction methods [12,13]. However, the SSST remains limited, as it can only be applied once per participant. In contrast, the proposed IMPRO protocol introduces a simple algorithm designed to generate reusable unpredictability, effectively overcoming this constraint.

### 1.1. Study Motivation

The Interactive Multitask Performance Response Observation protocol presents a novel approach to acute stress induction, designed to bridge the gap between artificial laboratory stressors and real-world cognitive–social challenges. The proposed protocol is centered around spontaneous speech improvisation, which demands real-time cognitive adaptability, creativity, and sustained verbal fluency under pressure. This difference in task structure makes IMPRO a more dynamic stressor than traditional methods. Unlike the TSST, which involves delivering pre-structured performance, IMPRO requires continuous speech generation in an unpredictable environment, where participants must integrate unexpected stimuli (random words) and solve cognitive tasks on the spot. This unpredictability mimics real-world high-stakes scenarios, such as negotiations or live discussions, making it highly transferable to natural settings.

In addition, the IMPRO protocol incorporates a gamification element to provide an engaging yet challenging experience. Rather than merely exposing participants to uncomfortable social threats, as in the TSST, IMPRO places participants in an interactive, problem-solving setting that induces acute stress while being engaged in cognitively challenging tasks. This aspect is particularly valuable for studies exploring stress resilience, executive function, and creative cognition in high-pressure environments.

By combining spontaneous performance with cognitive load, IMPRO offers a low-cost, easy-to-administer, and scalable alternative to traditional stress tests, with applications in neuroscience, psychology, and human performance research.

### 1.2. Study Objectives

This study designs the IMPRO protocol and validates its effectiveness in inducing acute stress by assessing markers including salivary cortisol and electrodermal activity. Salivary cortisol, a widely recognized biochemical marker of HPA-axis activation, serves as the gold standard for stress assessments [14]. EDA, recorded via a research-grade wearable wristband, provides insight into autonomic nervous system responses [15,16].

We hypothesize that IMPRO evokes a physiological stress response comparable to established paradigms. By utilizing objective physiological data, this study aims to position IMPRO as a scalable, ecologically relevant tool for acute stress research, with potential applications across neuroscience, psychology, and behavioral sciences.

### 1.3. Hypotheses

**H1.**  *The IMPRO protocol will induce a statistically significant increase in salivary cortisol levels from baseline to post-task, indicating activation of the HPA axis in response to acute stress*.

**H2.**  *The IMPRO protocol will lead to a significant increase in electrodermal activity during the stress-induction phase, with peak responses occurring during cognitively demanding tasks (Tasks B and C), followed by a gradual decline during the recovery phase*.

**H3.**  *The IMPRO protocol will serve as a feasible and ecologically valid alternative to traditional stress paradigms by evoking physiological stress responses while maintaining participant engagement, supporting its applicability in future stress research and experimental psychology*.

## 2. Materials and Methods

### 2.1. Ethics Statement

This study was approved by the Ethics Review Board at the Kyoto University of Advanced Science (approval number 23E03, date of approval 13 July 2023). All participants signed written informed consent form before taking part in the experiment.

### 2.2. Participants

To be eligible for this study, participants had to be healthy adults aged 18 to 35, fluent in English, with normal hearing, and with no history of neurological or psychiatric disorders. No formal assessments of reading or speaking fluency were conducted prior to testing; language proficiency was determined based on a self-report and academic enrollment in English-based academic programs.

Exclusion criteria included chronic illnesses, current medication affecting cortisol levels or cognitive function, substance abuse, and recent major life stressors. Recruitment took place from 13 June 2023 to 15 February 2024 through campus advertisements, flyers, and word-of-mouth referrals from colleagues. A total of 44 applicants signed up, of whom 37 met the eligibility criteria and were enrolled in the study. This sample size is consistent with previous studies validating acute stress protocols using physiological and hormonal measures and was considered sufficient to detect moderate within-subject effects [6,17].

Two participants were excluded from the statistical analysis due to poor data quality. The demographic information of the participants is presented in Table 1. The detailed age distribution is presented in Figure S1. Participants were compensated with an Amazon gift card (approximately US $20) as a token of appreciation for their time and efforts.

### 2.3. Study Procedure

**Figure 1:**
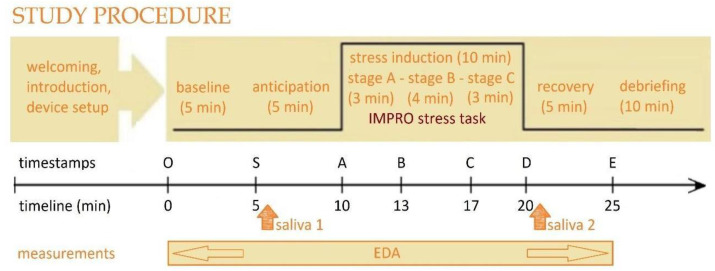
Study procedure. The experimental protocol consisted of six sequential stages: (1) welcoming and device setup, (2) baseline resting (5 min), (3) anticipation phase (5 min), (4) stress induction using the IMPRO protocol (10 min, consisting of three tasks: Stage A = 3 min, Stage B = 4 min, Stage C = 3 min), (5) recovery phase (5 min), and (6) debriefing (10 min). Salivary cortisol samples were collected at two time points: before the stress task (Saliva 1 at 5 min) and after the stress task (Saliva 2 at 20 min). Electrodermal activity was continuously recorded throughout the session, from the baseline period to the recovery phase. The timeline below indicates the experimental timestamps in minutes, with key events marked as O (start), S (baseline), A (anticipation), B (task initiation), C (mid-task), D (end of task), and E (end of recovery).

This study used a within-subject laboratory-based design to validate the IMPRO protocol in inducing acute stress. As shown in Figure 1, the experiment procedure consisted of six stages: (1) welcoming and device setup, (2) baseline awake resting (O–S), (3) anticipation (S–A), (4) stress induction using the IMPRO protocol (A–D), (5) stress recovery (D–E), and (6) debriefing. To assess the cumulative effect of the IMPRO tasks, salivary cortisol levels were measured and compared to the baseline. In addition, EDA was recorded and analyzed to evaluate the cumulative impact of the IMPRO tasks relative to baseline and recovery, as well as the dynamic changes between stages (Table 2).

#### 2.3.1. Setup and Baseline

The experiment began with a 15 min preparation stage, during which the researcher welcomed the participants, provided an overview of the study, and ensured their comfort. The participants were then asked to sign an informed consent form, complete all necessary paperwork, and wear wristbands on both hands. They had been informed beforehand about the tasks they were supposed to perform.

Following this, the awake resting stage began, where the participant sat quietly for 5 min to establish a baseline. Next, in the anticipation stage, which also lasted 5 min, the participant received detailed instructions about the upcoming improvisation tasks and had the opportunity to ask clarifying questions or prepare their thoughts in silence.

#### 2.3.2. Stress Induction

Acute stress induction was achieved through a series of improvisation tasks, which lasted 10 min. The proposed protocol was presented to participants as a game. The game consists of three tasks with increasing levels of difficulty: free improvisation (Task A), random words challenge (Task B), and an arithmetic load (Task C). Two human listeners were physically present in the experimental room throughout these tasks, seated silently in front of the participant. They acted as a live, non-responsive audience, refraining from any non-verbal feedback (e.g., smiling, nodding), to maintain a socially evaluative yet neutral setting. Video recordings of the sessions were made for analysis.

**Task A** requires the participant to speak without pause on any free topic for 3 min. If the participant pauses for more than 5 s, the task resets to the beginning. The purpose of this task is to allow participants to settle into their performance strategy and warm up.

**Task B** extends the improvisation with a new challenge. This task lasts 4 min, during which the listeners “throw in” random words, and the speaker must incorporate these words into the narrative immediately, without pausing to think. The goal of this task is to induce verbal confusion. This is a divergent task with no right or wrong answer, and participants can choose from various strategies for interacting with the new words. Participants are not informed of the words in advance and must adapt their strategy during the task. The researcher takes notes on the participant’s strategy (e.g., formal repetition, integration in the narrative, or ignoring the word). It is worth noting that ignoring the words did not result in the game being stopped as long as the participant continued speaking.

**Task C** is similar to Task B but with a slightly different challenge and lasts 3 min. The audience “throws in” arithmetic problems, and the participant must answer each one immediately and then continue the speech without pausing. This task introduces a convergent stress factor, as there is only one correct answer. Incorrect answers or ignoring the problems do not result in stopping the game, and the audience does not comment on the accuracy of the answers. A further stress factor across all three tasks is that the audience is not allowed to show any non-verbal support or approval, such as smiling or nodding.

#### 2.3.3. Recovery

After completing all three tasks, participants relaxed in a comfortable chair for 5 min. During the recovery stage, participants were encouraged to sit in a more relaxed position while having a casual conversation with the lead researcher. They were involved in a brief, semi-structured interview to discuss their subjective experience during the tasks. Sample questions included the following: “How was it for you?”, “What did you feel?”, “What was difficult?”, “What stood out to you?”. This stage served as a second control period, allowing for a comparison between improvisation speech and casual conversation.

Finally, in the 10 min debriefing phase, the researcher removed the measurement devices, provided feedback to the participant, and addressed any questions about the study. The researcher also collected feedback on the experiment design, the comfort and functionality of the measurement devices, the participants’ interactions with the listeners, and their overall impression of the study.

#### 2.3.4. Justification of Task Duration and Sequence

The IMPRO protocol was designed to gradually increase the cognitive and emotional load through a structured sequence of tasks within improvisational settings. The 3-4-3 min sequence allows participants to adapt to the task demands while experiencing increasing cognitive pressure. Task A (free improvisation) provides a low-pressure warm-up phase, allowing participants to familiarize themselves with speaking continuously. Task B (random words challenge) introduces unexpected cognitive interference, requiring participants to process and integrate external stimuli while maintaining fluent speech. Task C (arithmetic load) introduces an additional cognitive burden, forcing participants to engage in dual-task processing [18]. Throughout the tasks, the presence of passive, non-responsive listeners added a social–evaluative stressor, known to amplify speaker anxiety [19]. The sequence and duration were carefully designed to ensure a balance between cognitive challenge, social pressure, and sustained engagement while preventing overwhelming distress.

### 2.4. Measurements

To validate the acute stress response to the IMPRO protocol, we analyzed changes in physiological markers. We compared cortisol levels—the gold-standard biochemical marker of stress—before and after the stress-induction tasks. We also measured EDA to assess sympathetic arousal. All measurements were time-synchronized to ensure data alignment.

#### 2.4.1. Salivary Cortisol

Cortisol samples were collected at two time points: (1) before stress induction (baseline) and (2) immediately after stress induction. Cortisol concentrations were measured using the SOMA Cube system (SOMA Bioscience, Oxford, UK), a validated diagnostic tool. The system utilizes lateral flow immunochromatographic strip (ICS) technology, which has demonstrated results comparable to ELISA-based laboratory assays [20]. The SOMA Reader measured the line intensity and converted these values into the corresponding cortisol concentrations in the saliva sample (nM, nanomolar) using a proprietary algorithm. This tool was chosen for its reasonable accuracy, rapid and cost-effective analysis, and suitability for high-throughput assessments, making it well-suited for capturing dynamic stress responses in naturalistic conditions [20]. To minimize circadian variation, cortisol samples were collected between 2:00 and 4:00 p.m.

#### 2.4.2. EDA Measurement and Preprocessing

EDA was recorded using the Empatica E4 (Empatica Inc., Cambridge, MA, USA) wristband, an FDA-approved research-grade device, on both wrists at a 4 Hz sampling rate. Data were collected noninvasively through skin contact. Participants pressed the time-stamp button on the wristband, following the researcher’s instructions. EDA measurements were recorded in microsiemens (μS). Raw data were collected using complementary software and further imported for preprocessing and statistical analysis, for which Python 3.10 was used.

**Preprocessing:** As an initial step, a low-pass Butterworth filter (0.5 Hz cutoff) was applied to remove high-frequency noise and attenuate the phasic component of the EDA signal. The further smoothing is achieved through a moving average window to isolate the tonic component [21]. To achive comparability across individuals, EDA signals were normalized using min–max scaling across the entire session, including baseline period. This normalization step prevents individual differences in baseline levels from confounding the results.

The analysis focused solely on the tonic component of EDA, also referred to as the skin conductance level (SCL). To extract SCL, a moving average filter with a one-minute time window was applied, ensuring the retention of slow, sustained changes in sympathetic arousal while minimizing high-frequency fluctuations. This approach was selected to balance signal stability and temporal resolution, minimizing noise while capturing meaningful autonomic changes for reliable comparisons across experimental stages. Given the IMPRO protocol’s emphasis on sustained cognitive and social stress, we prioritized the tonic component of EDA as a primary measure of the acute stress response. Monitoring the tonic component enables the effective tracking of gradual changes in sympathetic arousal over extended periods [22], making it relevant for capturing stress accumulation across the anticipation, stress induction, and recovery phases. Unlike the phasic component, which reflects transient responses to discrete stimuli, tonic EDA provides a stable measure of overall autonomic activation, minimizing the variability introduced by task-specific fluctuations. This allows for a robust comparison of sympathetic dynamics across all stages.

**Data Synchronization:** The Synchronization of the Empatica E4 and SOMA-Cube measurements was achieved by timestamping each event using synchronized system clocks and manual markers (button presses) at key experimental time stamps (O, S, A, B, C, D, E) to separate each experimental stage. The following stages were indicated: OS “baseline” (5 min); SA “anticipation” (5 min); AB “Task-A” (3 min); BC “Task-B” (4 min); CD “Task-C” (3 min); DE “recovery” (5 min).

### 2.5. Statistical Analysis

Data distributions were tested for normality using the Shapiro–Wilk test. Paired *t*-tests were used to compare cortisol levels before and after the acute stress induction. For EDA analysis, repeated-measures ANOVA was performed to assess changes in autonomic activity across experimental stages (5th, 10th, 13th, 17th, 20th, 25th minute). Data for ANOVA were extracted from the final one-minute interval of each stage to minimize transient fluctuations and capture stable tonic EDA responses, which better reflect gradual changes in sympathetic arousal. Mauchly’s test of sphericity was performed, and the Greenhouse–Geisser correction was applied where sphericity was violated. Mean EDA values were compared across stages, and polynomial trend analyses (linear, quadratic, cubic) were conducted to evaluate temporal dynamics. Effect sizes (Cohen’s d, partial η^2^) were reported for all primary comparisons. All statistical analyses were performed with the significance threshold set at α = 0.05. Parametric tests were used to evaluate group differences and within-subject effects, with appropriate corrections applied for assumption violations (e.g., Greenhouse–Geisser for sphericity). Effect sizes, including Cohen’s d and partial η^2^, were reported for all key comparisons. Where applicable, Bonferroni correction was used to adjust for multiple comparisons.

## 3. Results

### 3.1. Cortisol Analysis

**Figure 2:**
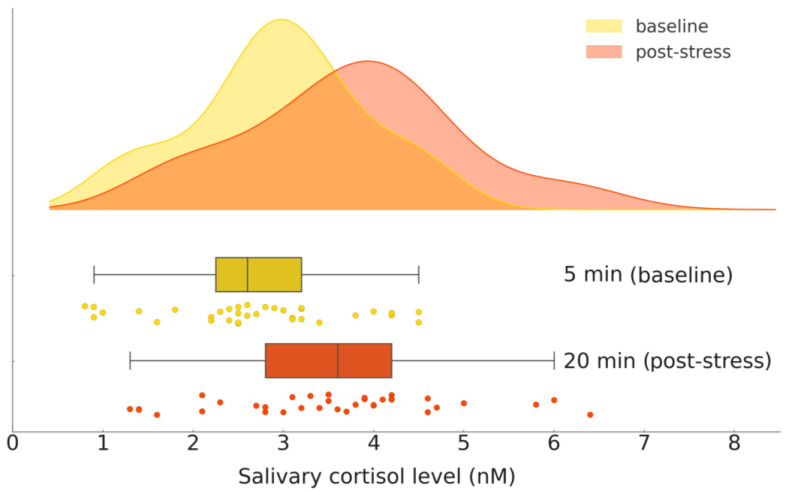
Salivary cortisol levels before (baseline) and after (post-stress) stress induction: raincloud plot.

A paired-samples *t*-test was conducted to compare cortisol levels at baseline (pre-task) and after stress induction (post-task). The Shapiro–Wilk test confirmed that the data were normally distributed (pre-task: W = 0.960, *p* = 0.229; post-task: W = 0.973, *p* = 0.516). Cortisol levels increased from pre-task (M = 2.68, SD = 0.99) to post-task (M = 3.54, SD = 1.25). The paired t-test showed a significant increase (t (34) = −3.488, *p* = 0.001), with a mean difference of 0.86 nM (95% CI [−1.36, −0.36]). The effect size (Cohen’s d = 0.59) indicates a moderate cortisol increase (Table 3). A boxplot (Figure 2) visualizes the distribution of cortisol values at each time point, highlighting the overall increase and individual variability.

### 3.2. EDA Analysis

Repeated measures ANOVA examined changes in EDA across experimental stages (at 5th, 10th, 13th, 17th, 20th, and 25th minute). Mauchly’s test of sphericity indicated a violation of the sphericity assumption (W = 0.074, χ^2^ (14) = 83.738, *p* < 0.001), requiring the application of the Greenhouse–Geisser correction (ϵ = 0.548). The analysis revealed a significant main effect of time on EDA levels, F (2.740,93.149) = 61.193, *p* < 0.001, indicating significant fluctuations in sympathetic arousal throughout the experiment. The effect size was large (η^2^ = 0.643), supporting the robustness of the observed changes. Bonferroni-corrected pairwise comparisons demonstrated significant increases in EDA from the 5th to 10th minute (*p* < 0.001) and from the 10th to 13th minute (*p* < 0.001), indicating a progressive sympathetic response both to anticipation and the initial stage of stress induction. EDA remained stable between the 13th and 17th minute (*p* = 0.075), suggesting a plateau phase. A significant decrease was observed from the 20th to 25th minute (*p* < 0.001), reflecting the recovery stage.

**Figure 3:**
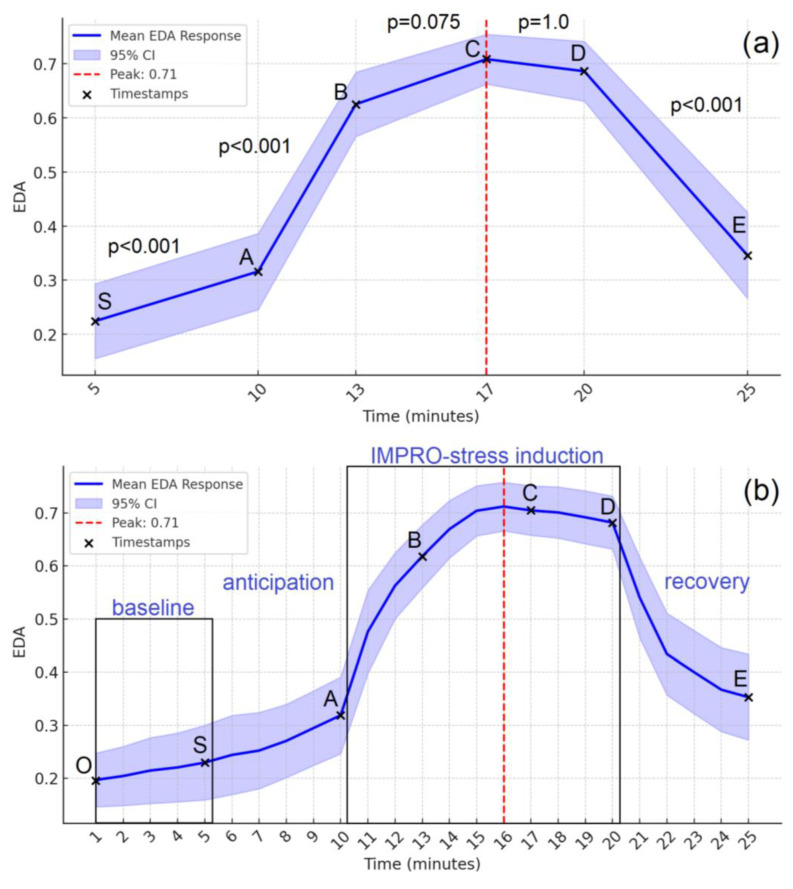
Electrodermal activity trajectory over time. Mean EDA responses are shown with 95% confidence intervals (CIs) across the experimental timeline. Signals were normalized (0–1 scale), including the baseline, and analyzed using a one-minute time window. (**a**) EDA changes at key timestamps (S, A, B, C, D, E) with Bonferroni-corrected pairwise comparisons. The red dashed line marks the peak response. (**b**) Full EDA trajectory across the baseline, anticipation, stress induction (IMPRO tasks A, B, C), and recovery. Timestamps (O–E) mark critical experimental phases.

**Figure 4:**
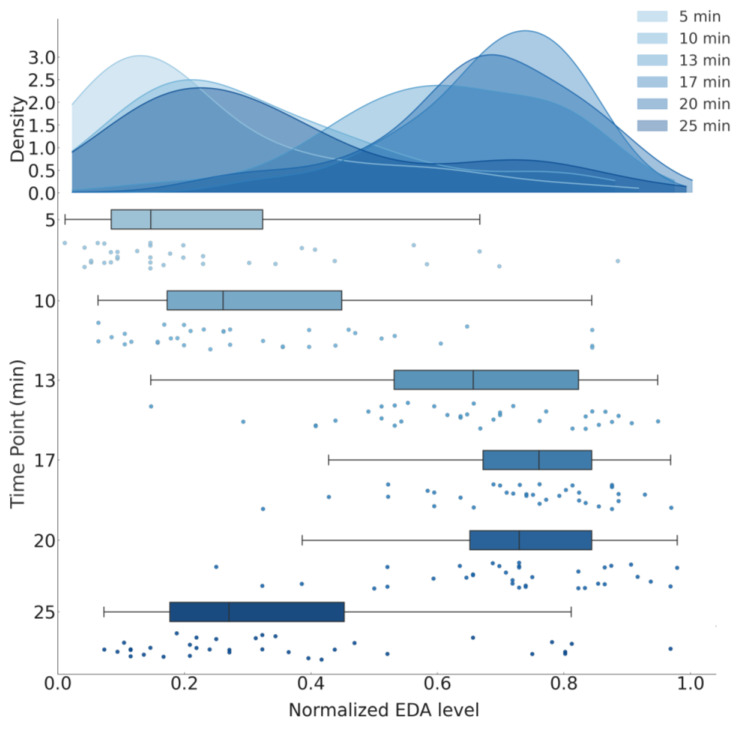
Temporal dynamics of electrodermal activity during stress and recovery: raincloud plot. The figure shows individual EDA values as jittered dots, overlaid with probability density estimates, interquartile range boxes, and median lines.

Trend analysis revealed a significant linear trend in EDA changes (F (1,34) = 64.816, *p* < 0.001), indicating a general increase and subsequent decrease in autonomic activity. Furthermore, a quadratic trend (F (1,34) = 91.488, *p* < 0.001) and a cubic trend (F (1,34) = 43.810, *p* < 0.001) were significant, reflecting non-linear dynamics in EDA responses, likely due to the stress induction and recovery processes. Figure 3 illustrates the EDA trajectory across time points, highlighting the peak response and subsequent recovery decline. The main results are presented in Table 4. Specifically, Figure 3a highlights key time points where significant EDA changes occurred, with statistical comparisons showing distinct differences between phases. Figure 3b provides a continuous view of EDA fluctuations across the entire experiment, illustrating a steady increase during stress induction, peaking at C, and gradually decreasing during recovery. Figure 4, Figure S2 and Figure S3 represent details of EDA temporal dynamics. These results demonstrate an autonomic stress response, followed by a return to baseline.

## 4. Discussion

### 4.1. Validity of IMPRO Stress-Induction Protocol

The reliability of the IMPRO protocol as an acute stress-induction method is supported by its consistent physiological effects across participants, as demonstrated by robust increases in salivary cortisol and electrodermal activity. The protocol elicited a significant post-task rise in cortisol levels (M = 3.54 nM, SD = 1.25) compared to baseline (M = 2.68 nM, SD = 0.99), confirming hypothalamic–pituitary–adrenal axis activation, a key marker of the stress response. These results align with prior research validating cortisol reactivity in stress paradigms, such as the Trier Social Stress Test, which typically induces a two- to three-fold cortisol increase in 70–80% of healthy adult participants [8,23].

Moreover, the EDA response trajectory observed with the IMPRO protocol follows a typical stress pattern, with a sharp increase during the anticipation and free improvisation (Task-A) stages, a plateau phase during peak cognitive load (Task B–C), and a significant decrease during recovery. The large effect size (η^2^ = 0.643) for EDA changes further supports its sensitivity to autonomic nervous system activation. These findings are in line with results from other validated acute stress paradigms that induce EDA reactivity, such as the Sing-a-Song Stress Test [12] and the Mannheim Multicomponent Stress Test (MMST) [24].

Together, cortisol and EDA provide complementary insights into the physiological effects of speech improvisation on performers. Cortisol indicates the endocrine stress response, influencing processes such as metabolism, immune function, and various bodily processes. In contrast, EDA reflects immediate sympathetic changes related to emotional arousal and attentional focus [17]. Using both markers allows us to gain a comprehensive understanding of how stress manifests both hormonally and in terms of autonomic nervous system activation following acute stress induction by the IMPRO protocol.

In addition to its validity, our IMPRO protocol has several advantages compared to existing stress-induction protocols. In terms of in-lab implementation, the IMPRO protocol is less resource-intensive than the TSST. It requires minimal preparation and fewer human resources, making it more accessible for a variety of research settings. This ease of use is particularly beneficial for studies with limited resources or those conducted outside of a laboratory environment.

Moreover, the protocol allows for customization based on the specific needs of different research populations and goals. The improvisational tasks can be easily adjusted to suit various age groups, cognitive profiles, and verbal fluencies, making it potentially suitable for a broad range of participants. For example, tasks can be tailored with age-appropriate scenarios for children and adults or structured prompts for neurodivergent individuals. Further, task difficulty and structure can be modified to address specific research questions. For example, increasing the number and frequency of random words can assess how well participants make decisions under pressure, while varying the audience size (e.g., small versus large group) can help explore the effects of social anxiety on stress responses.

An important feature of our IMPRO protocol is the gamification of stress-induction tasks. By framing the stressor as a game, the IMPRO protocol provokes eustress—a positive form of stress, as evidenced by 28 out of the 35 participants who reported in the interviews that the stress-induction tasks were both challenging and enjoyable. As such, our protocol maintains the physiological response associated with stress while minimizing the potential negative emotional impact, such as anxiety or emotional vulnerability, that might otherwise arise from traditional stress-induction protocols. These benefits could position the IMPRO protocol as a less invasive and ethically more acceptable alternative to existing methods.

While a direct comparison with established protocols like the TSST was beyond the scope of this study, this remains an important avenue for future research. Further studies should explore how the IMPRO protocol compares to traditional methods, particularly in terms of its ability to elicit physiological stress responses while reducing negative emotional impact.

### 4.2. Scalability and Group Extension

While the IMPRO protocol was originally designed for individual acute stress induction, it holds great potential for scalability to larger cohorts by extending to group-based settings. Adapting the protocol for group use would offer a resource-efficient approach to stress research, similar to the adaptations made in the Trier Social Stress Test for Groups [9]. In a group-based IMPRO setup, multiple participants could engage in simultaneous or sequential improvisational tasks, with some giving speeches while others act as the audience. Logistically, a group-based design would reduce the session time per participant while maintaining the stress-inducing nature of the task, making it more feasible for larger-scale studies. Future research could explore how group dynamics influence stress responses, particularly in the context of interpersonal interactions, emotional contagion, and collective stress resilience.

### 4.3. Future Directions

While the present study focused on validating the physiological impact of the IMPRO protocol using established frequentist methods, we acknowledge that Bayesian inference offers a complementary framework for evaluating the evidence strength. Incorporating Bayes factors in future analyses may enhance the interpretability of results, particularly in cases with small to moderate effect sizes or non-significant trends, by quantifying relative support for alternative versus null hypotheses.

In addition to physiological and hormonal measures, future research should incorporate behavioral performance metrics during improvisation tasks. Metrics such as precision and recall in response to task prompts could offer a more granular understanding of participant strategies, engagement, and individual differences in stress reactivity. Performance analyses would complement physiological data and provide a more comprehensive validation of the protocol.

### 4.4. Limitations

Despite the promising results, this study has several limitations. Firstly, the protocol involves various interventions and cognitive processes, including social stress, cognitive load, speech production, and improvisation, all of which require spontaneous performance without prior preparation. This multifaceted approach makes it difficult to isolate the specific contributions of each factor to the overall stress response. Secondly, the study did not explore the dynamics of stress markers during the post-intervention period. Third, the study sample consists primarily of university students. This limitation may impact the generalizability of the findings to a broader population, including different age groups and cultural backgrounds.

Importantly, participants were not formally assessed for verbal fluency or speaking skills prior to the task, which may have influenced performance and stress reactivity under improvisational conditions. Similarly, individual differences in stress resilience, state/trait anxiety, and prior experience with public speaking were not measured, potentially contributing to inter-individual variability in physiological responses. These unmeasured psychological factors should be accounted for in future studies using validated self-report instruments or behavioral assays.

Moreover, while the dataset included both male and female participants, the sample size was not sufficiently powered to explore sex-based differences in cortisol and EDA responses through subgroup analysis. Future studies with larger, stratified samples should examine demographic factors.

Lastly, the present study did not conduct a multivariate analysis to investigate the joint effects of multiple physiological features. Such approaches may offer deeper insight into the complex interplay between autonomic and endocrine markers and are recommended for future analyses.

## 5. Conclusions

This study validates the proposed IMPRO protocol as an effective tool for inducing acute stress. The significant increases in both salivary cortisol levels and EDA demonstrate that the protocol successfully engages both the HPA axis and the autonomic nervous system, validating its potential as a reliable method for stress induction. Future research should focus on evaluating the ecological validity of IMPRO, examining its relevance and applicability in real-world settings. Comparative studies are also needed to assess the effectiveness of IMPRO relative to existing stress-induction methods. Finally, investigating the feasibility and validity of adapting the protocol for group formats could further expand its potential for use in both research and clinical settings.

## Figures and Tables

**Table 1:** Demographic information.

Demographic	Description		Number	Percentage
Age	18–23 subgroup	19.4 (1.3) years	21	60.00%
	24–29 subgroup	25.6 (1.4) years	8	22.86%
	30–38 subgroup	31.8 (1.9) years	6	17.14%
	Total	23.0 (5.0) years	35	100.00%
Gender	Male		19	54.29%
	Female		16	45.71%
	Total		35	100.00%
Native language	**Language Family:**	**Language** **with branch:**		
	Indo-European	Russian (Slavic branch)	5	14.28%
		Ukrainian (Slavic branch)	2	5.71%
		Spanish (Romance branch)	3	8.57%
		Portuguese (Romance branch)	1	2.86%
		French (Romance branch)	1	2.86%
		English (Germanic branch)	3	8.57%
		German (Germanic branch)	1	2.86%
		Hindi (Indo-Aryan branch)	2	5.71%
		Urdu (Indo-Aryan branch)	2	5.71%
		Bengali (Indo-Aryan branch)	3	8.57%
	Dravidian	Tamil	1	2.86%
	Turkic	Azerbaijani	1	2.86%
		Mongolian *	1	2.86%
	Kartvelian	Georgian	1	2.86%
	Sino-Tibetan	Sinhala	4	11.42%
	Kra-Dai	Thai	1	2.86%
	Austronesian	Indonesian	1	2.86%
	Niger-Congo	Swahili	1	2.86%
	Japanese	Japanese	1	2.86%
	Total		35	100.00%
Educational level	Bachelor students (1st–2nd year)		12	25.71%
	Bachelor students (3rd–4th year)		8	31.43%
	Master students		7	20%
	PhD students		4	11.43%
	Other		4	11.43%
	Total		35	100.00%
English proficiency level	Native		3	8.57%
	Advanced		28	80%
	Upper intermediate		4	11.43%
	Total		35	100.00%
Munich Chronotype Questionnaire (MCTQ)	Morning		3	8.57%
	Evening		24	68.57%
	Night		8	22.86%
	Total		35	100.00%
Perceived stress scale (PSS)	Low		9	25.71%
	Moderate		20	57.14%
	High		6	17.14%
	Total		35	100.00%

* Though sometimes categorized separately, the Mongolian language is sometimes grouped with the Turkic language family due to historical influences.

**Table 2:** Study procedure. The timeline of the study and key measurement points.

Stage		Time	Code	Description
Preparation		15 min	-	Participants were welcomed, briefed on the study, and equipped with measurement devices.
**➡** **Start EDA measurements**				
Baseline		5 min	O–S	Participants sat quietly to establish physiological baselines.
**➡** **Saliva-1 (collection of the first saliva sample)**				
Anticipation		5 min	S–A	Participants received task instructions and prepared for performance.
IMPRO stress induction	Task A: Free improvisation	3 min	A–B	Continuous speech without pauses.
	Task B: Random words challenge	4 min	B–C	Speech improvisation with unexpected words introduced by an audience.
	Task C: Arithmetic load	3 min	C–D	Speech improvisation with real-time arithmetic challenges.
**➡** **Saliva-2 (collection of the second saliva sample)**				
Recovery		5 min	D–E	Participants relaxed while engaging in informal conversation with the researcher.
**➡** **Stop EDA measurements**				
Debriefing		10 min	-	Device removal. Feedback collection

**Table 3:** Cortisol analysis with paired samples *t*-test.

Condition	95% CI	Min–Max	Median	IQR
Pre-Task (5 min)	2.35–3.03	0.80–4.50	2.60	1.00
Post-Task (20 min)	3.12–3.98	1.30–6.40	3.60	1.40

**Table 4:** Electrodermal activity analysis with repeated-measures ANOVA.

Condition (min)	95% CI	Min–Max	Median	IQR
5th (baseline)	0.482–0.560	0.310–0.740	0.525	0.142
10th (anticipation)	0.568–0.658	0.401–0.890	0.610	0.156
13th (task-A)	0.651–0.753	0.470–0.975	0.705	0.165
17th (task-B)	0.633–0.735	0.455–0.960	0.680	0.159
20th (task-C)	0.623–0.719	0.439–0.915	0.670	0.150
25th (recovery)	0.543–0.635	0.385–0.812	0.590	0.145

## Data Availability

Due to the sensitive nature of the human participant data, the datasets are not publicly available. Data can be provided by the corresponding author upon reasonable request, subject to ethical approval.

## Supplementary Materials

The following supporting information can be downloaded at: https://www.mdpi.com/article/10.3390/brainsci15050522/s1, Figure S1: Participant age distribution by subgroup; Figure S2. Electrodermal activity across key time points; Figure S3. Mean electrodermal activity over time with 95% confidence Intervals.

## Abbreviations

The following abbreviations are used in this manuscript:

IMPRO	Multitask Performance Response Observation
TSST	Trier Social Stress Test
EDA	electrodermal activity
HPA	confirming hypothalamic–pituitary–adrenal (axis)
IMPRO	Multitask Performance Response Observation
TSST-G	TSST for Groups
SSST	Sing-a-Song Stress Test
MCTQ	Munich Chronotype Questionnaire
PSS	Perceived stress scale
ICS	flow immunochromatographic strip
ELISA	enzyme-linked immunosorbent assay
FDA	Food and Drug Administration
SCL	skin conductance level
ANOVA	analysis of variance
IQR	interquartile range
SD	standard deviation
CI	confidence interval
M	mean
nM	nanomolar (equivalent to nmol/L)
μS	microsiemens
