# Supplementary material for: Validation of a New Stress Induction Protocol Using Speech Improvisation (IMPRO)
Source: Brain Sci. 2025 May 19;15(5):522. doi: 10.3390/brainsci15050522 (PMC12110528; doi:10.3390/brainsci15050522)
Supplement: Supplementary file 1 [file brainsci-15-00522-s001.zip › brainsci-3638458-supplementary.pdf]

# Supplementary Materials

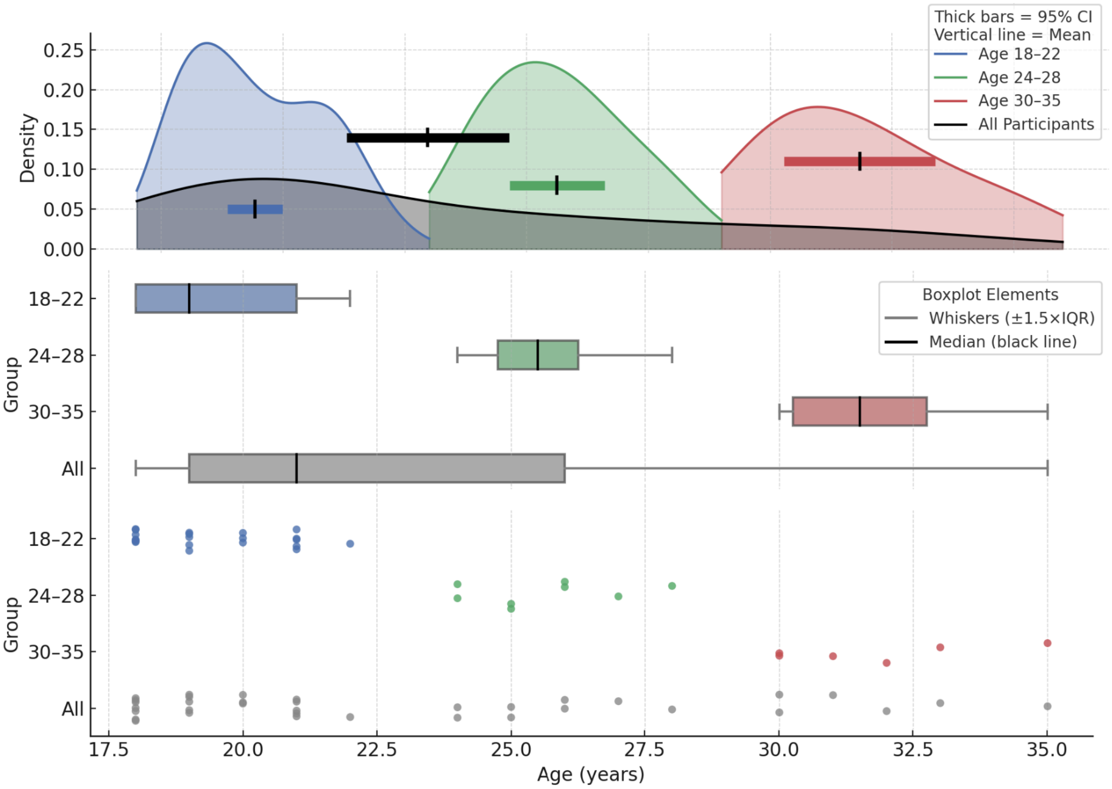


**Supplementary Figure S1. Participant age distribution by subgroup.** This figure presents a comprehensive summary of participant age across three predefined subgroups (18–22, 24–28, and 30–35 years), as well as the overall sample ("All").
**Top panel:** Kernel density estimates (KDE) show smoothed age distributions per subgroup, with overlaid thick bars representing 95% confidence intervals (CI95) around the group means.
**Middle panel:** Horizontal boxplots visualize the median, interquartile range (IQR), and whiskers (±1.5×IQR) of age distributions across groups.
**Bottom panel:** Jittered individual values highlighting within-group variability and distribution shape.

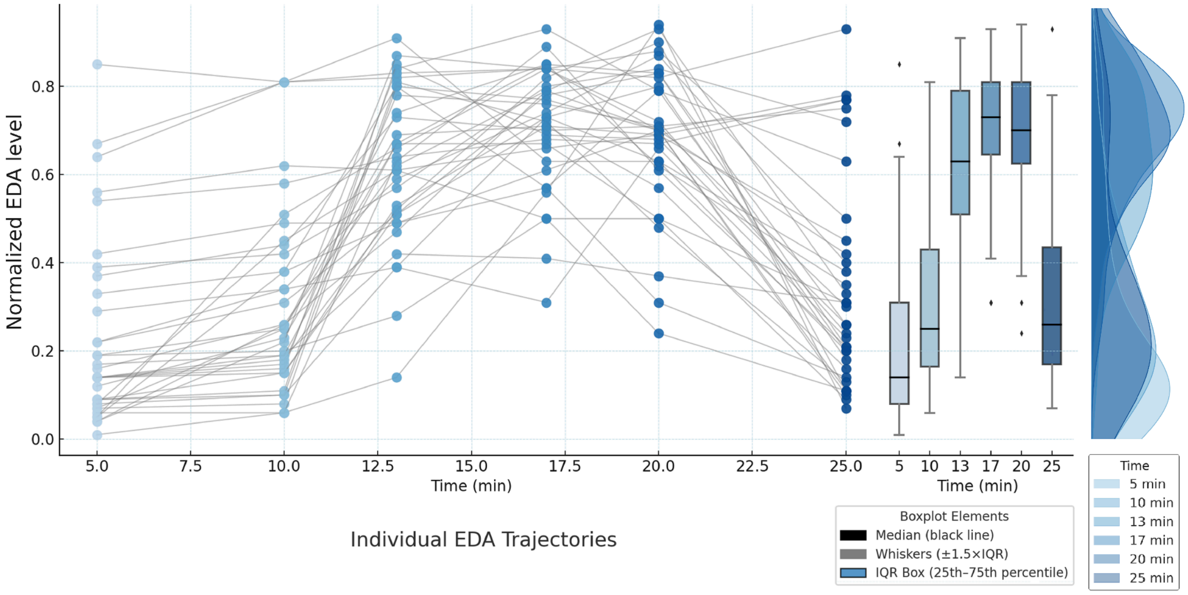


**Supplementary Figure S2. Electrodermal activity across key time points.** This figure illustrates within-subject dynamics of normalized electrodermal activity across six key experimental time points (5, 10, 13, 17, 20, and 25 minutes), capturing the progression of stress induction and recovery phases.

**Left panel:** Individual participant trajectories and values at each time point, shaded from light (5 min) to dark blue (25 min) to represent temporal progression.

**Center panel:** Boxplots summarize group-level EDA distributions. Boxes represent the interquartile range (IQR; 25th–75th percentile), black lines indicate medians, and whiskers extend to ±1.5×IQR. Outliers are displayed as individual points.

**Right panel**: Kernel density estimates (KDEs) show the smoothed distribution of normalized EDA values at each time point.

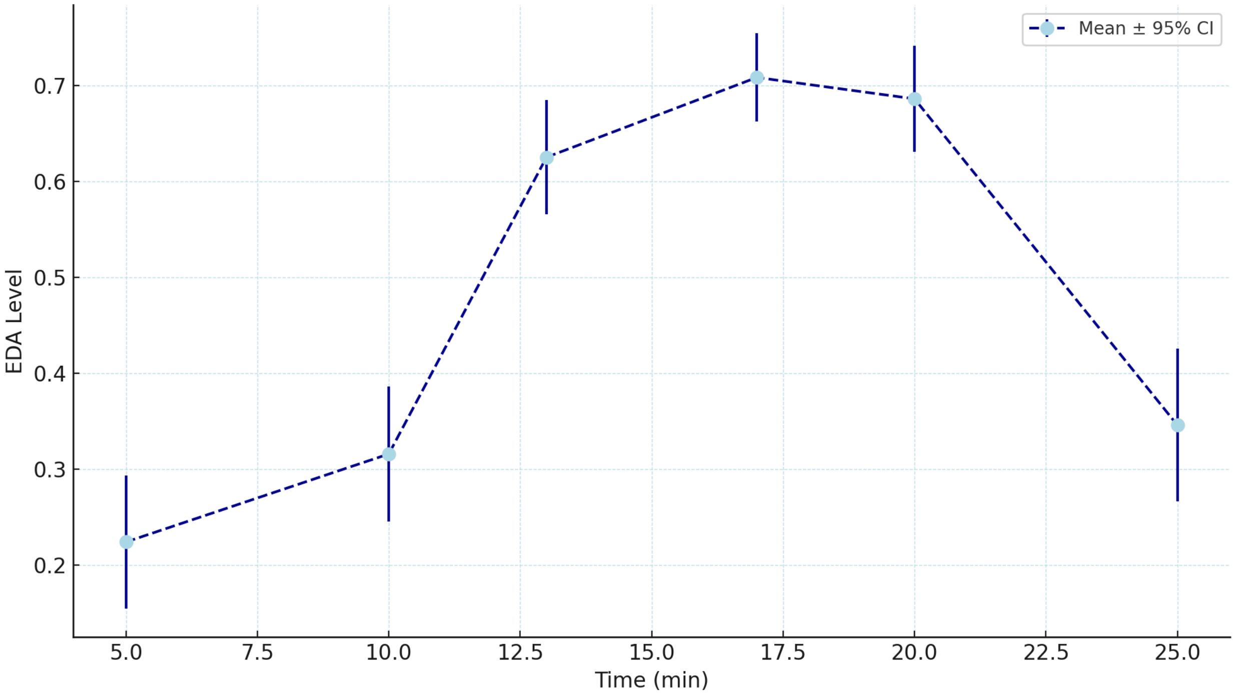


**Supplementary Figure S3. Mean electrodermal activity over time with 95% confidence intervals.** This figure presents the group-level trajectory of normalized electrodermal activity across six key time points (5, 10, 13, 17, 20, and 25 minutes), summarizing the temporal pattern of physiological arousal during the stress induction and recovery phases.
